# Monitoring Ovarian Stimulation for Assisted Reproduction With Patient Self-Scans Using a Home Vaginal Ultrasound Device: A Single-Center Interventional, Prospective Study

**DOI:** 10.2196/72607

**Published:** 2025-08-06

**Authors:** Yoel Shufaro, Mor Cohen, Avital Wertheimer, Eran Altman, Leor Wolff, Onit Sapir, Avi Ben-Haroush, Alyssa Hochberg

**Affiliations:** 1 Infertility and in vitro fertilization (IVF) Unit, Beilinson Hospital, Rabin Medical Center, 39 Jabotinsky Street, Petah Tikva, 4941492, Israel; 2 The Faculty of Medicine, Tel Aviv University, Ramat Aviv, Tel Aviv, Israel; 3 Translational Medical Innovation, eHealth and Digital Wing, Clalit Health Services, Tel Aviv, Israel

**Keywords:** in-vitro fertilization, follicle monitoring, self vaginal sonography, home sonography, mobile phone

## Abstract

**Background:**

Ovarian follicles and endometrial thickness are monitored repeatedly for assisted reproduction, burdening patients and clinics. Self-scans with a home ultrasound device can relieve this.

**Objective:**

We aimed to evaluate the reliability of self-scans using the smartphone-based Pulsenmore follicle count vaginal self-scan device (FC) versus in-clinic (IC) sonographies, in ovarian stimulation for in-vitro fertilization or fertility preservation.

**Methods:**

This study is a single-center, interventional, controlled, prospective study including 44 patients without pelvic pathologies undergoing stimulation for in-vitro fertilization (2022‐2024). Following training, patients used a vaginal home ultrasound device to scan their uterus and ovaries with remote guidance by a sonographer in each cycle check-point. Clinical decisions were based on standard IC sonographies. FC and IC results were compared for image quality, endometrial thickness, and follicle count or size. Aspirated oocyte numbers were compared to the follicles recorded at the last visit by home and IC scans. Absolute differences in follicular count and endometrial thickness between IC and FC scans were compared using means, SDs, and 95% CIs. The Spearman correlation (r) analyzed the relations between IC and FC outcomes. All tests applied were 2-tailed, with a *P* value of ≤5% considered statistically significant. Patient and sonographer satisfaction were assessed via surveys.

**Results:**

Of 44 patients, 34 completed this study. The mean age was 34.7 (SD 4.0) years, and BMI was 25.8 (SD 5.0) kg/m². A total of 65% (22/34) pursued fertility preservation and 35% (12/34) aimed to conceive. The image quality scores of all home scans were at a minimum suitable level, with most of better quality. FC measurements closely matched IC findings for key clinical parameters: antral follicle count (mean FC 11.94, SD 6.62 vs mean IC 15.23, SD 10.2, ρ=0.86, *P*<.001); number of stimulated follicles ≥10 mm (FC 12.19, SD 6.27 vs IC 13.5, SD 8.87, ρ=0.84, *P*<.001); identification of the leading follicle >14 mm (achieved in 87% of FC scans); and follicular number or size pretriggering. The aspirated oocyte or last-visit stimulated follicles (>10 mm; FC 1.12, SD 0.6 vs IC 1.06, SD 0.56, ρ=0.82, *P*<.001), mature oocytes or follicles >13 mm ratios (FC 1.28, SD 1.11 vs IC 1.04, SD 0.77, ρ=0.88, *P*<.001), and endometrial thickness pretriggering (FC 9.87, SD 2.2 mm vs IC 9.63, SD 2.7 mm, ρ=0.54, *P*=.002) were well-correlated between the home and standard scans, with 87.1% concordance in identifying endometrial adequacy (≥7 mm). In the patient survey, 82% (28/34) expressed interest in future use of the FC device. In the sonographer survey, 91% (31/34) demonstrated patient improvement.

**Conclusions:**

The home ultrasound device was feasible, comparable, and well-correlated with standard IC scans, laying the basis for remote home-based monitoring of follicular development during ovarian stimulation. We believe this also applies to monitoring milder stimulations and even natural cycles.

## Introduction

Follicle number and size are key parameters for monitoring ovarian stimulation during assisted reproduction cycles and for determining the time for oocyte maturation triggering and pick-up [1]. The sonographic endometrial thickness and pattern are important parameters associated with implantation and pregnancy [2]. Both are measured repeatedly in serial vaginal sonographies during the entire assisted reproduction cycle, imposing a significant burden of time, stress, travel, and cost on patients, requiring expensive in-clinic (IC) sonography facilities and adding to the clinic’s workload.

Patient self-scans with a home ultrasound device can potentially relieve this burden for both patients and clinicians. After proper training, capable and willing patients could scan their ovaries with a home vaginal probe connected to their smartphone screens. The scans are then uploaded to their clinic’s server and evaluated for the number of ovarian follicles and their diameter, as well as the endometrial thickness and pattern, just like any IC scan [3]. Patients have the liberty to perform the scans in the comfort of their homes, while clinicians can assist virtually to ensure the scans are complete and can measure the follicles and endometrium later on. Based on this sonographic information, together with blood hormone tests performed off-site, the ovarian stimulation can be monitored and managed. Recent studies aimed to integrate telemedicine into assisted reproduction, advancing its simplicity and accessibility to patients. Gerris et al [4] have explored in a step-wise manner the use of home ultrasonography by patients or their partners to perform transvaginal sonographies at their convenience, thereby significantly alleviating the cost and stress of monitoring [5]. The main outcomes demonstrated that patients are willing and capable of performing effective transvaginal home sonography, following appropriate training. This randomized controlled trial demonstrated that self-operated home sonography is noninferior compared to IC conventional sonography, with promising clinical outcomes and health care cost savings [4]. Additional studies also supported this notion, but with limitations of small sample sizes, minimal or partial follicular monitoring, and observational data, with no true comparison between home self-scans and IC monitoring [5-10].

The path to the vision of remote folliculometries is challenging concerning technical development and patient self-use. The vaginal probe should be robust and provide high image quality with rapid acquisition and have reasonable weight, interface, and price. It should be friendly for intimate pelvic use, and a patient-training protocol targeted at accomplishing complete self-scans in a domestic environment should be developed and evaluated.

In this study, we aimed to evaluate the real-life reliability and accuracy of patient self-scans using the smartphone-based Pulsenmore follicle count vaginal self-scan device (FC) in comparison to standard IC sonographies, in patients undergoing ovarian stimulation and oocyte pick-up (OPU) for assisted reproduction or fertility preservation. In addition to the “medical” objective outcomes, evaluation of the subjective patient experience and satisfaction was also in the scope of this study.

## Methods

### Study Design

This study is a single center, interventional, controlled, prospective study in women undergoing ovarian stimulation and OPU for in-vitro fertilization (IVF) or fertility preservation, designed to examine the safety and effectiveness of the FC device in comparison to conventional IC ultrasound measurements performed by a professional sonographer using a standard ultrasound device (GE Voluson E8 with a vaginal 9 MHz probe). This study was performed between December 2022 and July 2024.

### Ethical Considerations

The performance of this study was approved by the local institutional review board (RMC 21‐0271), and the study was registered at ClinicalTrials.gov (NCT05485623). All patients enrolled consented to participation in the study.

### Participants

The patient inclusion criteria were the following: ages 18‐43 years, normal pelvic anatomy, and capability and willingness to perform self-vaginal ultrasound scans and to operate the user interface. The exclusion criteria included: known uterine malformations or ovarian pathologies; expected diminished ovarian response according to the POSEIDON (Patient-Oriented Strategies Encompassing Individualized Oocyte Number) criteria [11]; presence of hydrosalpinx; intra-abdominal adhesions; previous lower abdominal surgery other than a cesarean; BMI >40 kg/m^2^; an allergy to the ultrasound probe, condom, or lubricant materials; and significant malposition of the ovaries.

### Study Protocol

**Figure 1.**
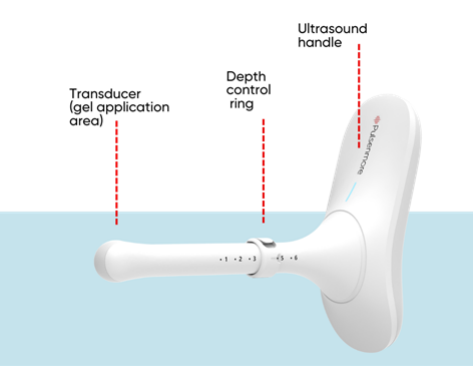
The Pulsenmore follicle count is a self-scan transvaginal ultrasound device, designed to be used in the comfort of one’s home. The device connects seamlessly with the patient’s personal smartphone, allowing them to perform transvaginal scans of the uterus and ovaries, with remote diagnosis provided by a qualified clinician. The depth control ring is set during the training session. FC: Pulsenmore follicle count home vaginal ultrasound.

Following recruitment and written informed consent, participants were provided with a personal FC device (Figure 1) with a smartphone for data transfer. They were trained in performing vaginal self-scans in the clinic by a professional sonographer according to the study protocol using the patient interface (see Multimedia Appendix 1). In brief, the participants were instructed by a sonography technician on how to operate the FC device and the smartphone application and were instructed in cleaning with the standard ultrasound condoms, lubricant, and wipers provided. Then the technician performed a guided vaginal ultrasound examination of the patients using the FC device connected to a smartphone, with her hands on theirs (guiding them with her hands placed on theirs). This examination was performed in a home-like setup, and not on an examination bed. The patients were instructed how deep to insert the vaginal probe and to which direction in order to identify the uterus in a midsagittal section and identify the endometrial lining. The depth ring was fixed on the probe, and the depth and gain of the FC device were optimized for the best visualization. Then the patients were instructed in the same manner how to visualize and scan each of their ovaries in the transverse section (see Multimedia Appendix 2). Following this hands-on demonstration, the patients were requested to perform the FC scan unassisted, while the technician observed the test from her computer and instructed the patient verbally only. The latter exam was already recorded and uploaded.

In the following meetings, the participants performed a self-scan of the uterus in a sagittal section and of the ovaries in a transverse section from their homes, with a remote online guidance by a professional sonographer, using the clinician interface on a distant computer. The FC examination was performed within 3 hours before or after each standard IC sonographic examination performed during the treatment cycle by a professional sonographer. Clinical decisions were based on the results of the standard IC sonographies performed in the clinic, including parameters such as the number of follicles and follicular sizes. The clips of the self-scans obtained with the FC device were analyzed separately for image adequacy, endometrial thickness, and number and diameter of the ovarian follicles. The measurements were performed manually by the sonographist who guided the patient online (not the same one who performed the IC examinations). The data obtained from the FC device scans were stored separately and independently from the patients’ records and analyzed outside the time frame of the treatment cycle. The quality of the images or clips obtained with the FC device was separately qualitatively assessed by a different sonographer according to the quality assurance grading scale [12]. Each patient participated in a single stimulation-OPU cycle. The participants and the sonographers assisting online and analyzing the FC scans were requested to fill separate user experience questionnaires, summing up their experience with the FC device and system.

The endometrial thickness, and number and diameter of ovarian follicles obtained in each FC device self-scan were compared to those obtained through the IC sonographies performed on the same day. The results of the last pretriggering visit of the FC device and the IC sonographies were compared to the outcomes of the OPU procedure, that is, the number of total and mature (metaphase II) oocytes. The IC measurements were performed using the standard device tool. The FC measurements were performed using the Pulsenmore interface.

The primary end points of the study were the safety of the FC device and the quality of the obtained images according to the quality assurance grading scale [12].

The secondary end points were the correlation between the FC device and IC scans in identifying the number of follicles >17 mm (within ±1 follicle difference) and the endometrial thickness (as ≥7 mm) during the last pretriggering visit. The correlation in the antral follicle count at the first visit, the total number of follicles at the last visit, and a comparison between the ratio of follicles to oocytes retrieved were defined as exploratory end points.

Patient satisfaction, a key parameter for successful adoption of home sonography, was defined as an additional separate outcome.

### Statistical Analyses

This study is small and preliminary in nature; therefore, the design of the analysis is descriptive, and the sample size was not planned to be statistically powered. Numerical variables were summarized using means and SDs. Categorical variables were summarized using frequencies and percentages. Differences in follicular count and endometrial thickness between IC and FC scans were compared with absolute differences using means, SDs, and 95% CIs. Relations between IC and FC outcomes were analyzed by the Spearman correlation (r) and using the Bland-Altman plot analysis. Root mean square difference (RMSD), absolute mean bias (AMB), and limits of agreement (LOA) were calculated for each parameter’s agreement assessment. To evaluate the patients’ dexterity in operating the FC device, the self-scan duration time difference between the first and last self-scans was calculated using the *t* test for dependent samples. All tests applied were 2-tailed, and a *P* value of 5% or less was considered statistically significant. The data was analyzed using R (version 4.4.1; R Development Core Team).

## Results

Forty-four eligible patients gave their consent to participate in the study. Five patients failed the initial training and screening, and 5 patients did not complete the study. Thirty-four patients completed this study, and full data of their FC scans were compared to the results of their IC scans. Their mean age was 34.7 (SD 3.99) years, and mean BMI was 25.8 (SD 5.05) kg/m^2^. A total of 65% (22/34) performed the treatment cycle for fertility preservation, and the remaining 35% (12/34) performed it to conceive. Among those treated for conception, unexplained infertility was the most prevalent diagnosis (20%).

**Figure 2.**
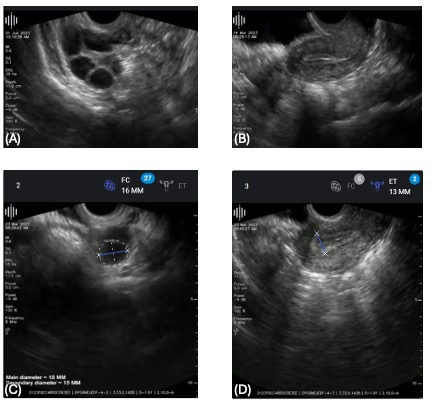
Demonstration of typical scans of (**A**) follicles and (**B**) the endometrium performed by the self-operated FC transvaginal device. Measurement tools are available in the clinician dashboard and are used for measurement of (**C**) follicle size and (**D**) endometrial thickness. FC: Pulsenmore follicle count home vaginal ultrasound.

The image quality score [12] of the FC scans was 3 for 37.1%, 4 for 29.7%, and 5 for 33.72%. No FC scan had an image quality score of 1 or 2 (none to minimally recognizable structures). Representative IC and FC images are shown in Figures2  3.

**Figure 3.**
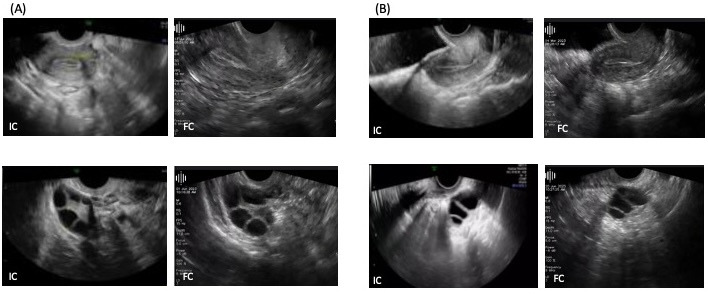
IC and FC scans taken from 2 representative patients on the same day ((A) patient A and (B) patient B). FC: Pulsenmore follicle count home vaginal ultrasound; IC: in-clinic.

The antral follicle count at the first visit was 11.94 (SD 6.62) for the FC scans, and 15.23 (SD 10.2) for the IC scans (absolute difference 4.19, 95% CI 1.42‐6.96, r=0.86, *P*<.001, RMSD 8.53; AMB 4.19; LOA −18.98 to 12.27). Correct identification of the first visit with a leading follicle ≥14 mm, in comparison to the IC scans as the gold standard, was achieved in 87.4% of the FC scans. The total number of stimulated follicles (≥10 mm) recorded from the FC scans was 12.19 (SD 6.27), and 13.5 (SD 8.87) from the IC scans (absolute difference 3.13, 95% CI 1.57‐4.68, r=0.84, *P*<.001; RMSD 9.94; AMB 4.86; LOA −22.22 to −14.67). Their subdivision into groups is detailed in Table 1 as: small (10-13 mm), intermediate (14-17 mm), and large (>17 mm), and shows a good correlation between FC and IC scan results interpretation, with a small absolute difference between them. In 90.6% of the last pretrigger scans, there was a difference of no more than ±1 large (>17 mm) follicle between the FC and IC scans. The endometrial thickness at the last pretrigger visit was 9.87 (SD 2.2) in the FC scans and 9.63 (SD 2.7) in the IC scans (absolute difference 1.7 mm, 95% CI 1.05‐2.39, r=0.54, *P*=.002; RMSD 2.49; AMB 1.72; LOA −4.7 to 5.17). In 87.1% of the last pretrigger visits, there was a correlation between the FC and IC scans in determining an endometrial thickness ≥7 mm, which is the threshold for endometrial adequacy [13].

**Table 1.** Number of follicles during the last pretrigger visit, as recorded from FC^a^ scans, in comparison to IC^b^ scans, divided according to diameter categories.

Follicles (mm)	FC, mean (SD)	IC, mean (SD)	Absolute difference	95% CI	Spearman correlation (ρ)	*P* value	Bland-Altman plot analysis (RMSD^c^/AMB^d^/LOA^e^)
10‐13	5.78 (4.68)	5.28 (5.19)	1.63	0.77‐2.48	0.82	<.001	2.84/1.62/(−5.07 to 6.07)
14‐17	5.03 (2.7)	6.28 (4.57)	1.938	0.75‐3.13	0.7	<.001	3.78/1.94/(−8.38 to 5.86)
>17	1.38 (2.01)	1.94 (2.26)	0.81	0.29‐1.33	0.69	<.001	1.64/0.81/(−3.63 to 2.5)

a FC: Pulsenmore follicle count home vaginal ultrasound.

b IC: in-clinic.

c RMSD: root mean square difference.

d AMB: absolute mean bias.

e LOA: limits of agreement.

The ratio of retrieved oocytes or number of follicles >10 mm at the last visit was mean 1.12 (SD 0.6) for FC scans and mean 1.06 (SD 0.56) for IC scans (absolute difference 0.21, 95% CI 0.11‐0.31, ρ=0.82, *P*<.001). The ratio of retrieved mature oocytes or number of follicles >13 mm at the last visit was 1.28 (SD 1.11) for FC scans and 1.04 (SD 0.77) for IC scans (absolute difference 0.39, 95% CI 0.12‐0.56, ρ=0.88, *P*<.001, RMSD 68.77; AMB 33.8; LOA −104.9 to 152.25).

The results of the patient experience survey at the completion of this study are presented in Table 2. All the patients who completed this study also completed the survey fully. A total of 82% (28/34) of participants were interested in further using the FC device in other cycles. The results of the sonographer experience survey are presented in Table 3. Full data about all patient home scans was obtained from the sonographers. In 91% (31/34) of patients, the sonographers noted patient improvement with cycle progression.

**Table 2.** Patient experience survey at the completion of the study (5=completely agree, 4=agree, 3=neutral, 2=disagree, and 1=completely disagree).

Question	Average score
I felt physically comfortable while using the home ultrasound device.	4.39
I did not experience any discomfort or pain beyond what is typical for a similar vaginal examination in the clinic	4.60
I got satisfactory training to perform self-scans	4.56
I trust myself to be able to perform self-scanning at home	4.11
I believe that the remote clinical monitoring is as reliable as the support I receive during clinical examination	4.04
In the future, I would be interested in adopting the Pulsenmore system for remote scanning at home during fertility treatment.	4.27
I’m interested and prefer to perform a self-scan at home since it is more comfortable and time-saving	3.88
I would recommend the use of the home device to other women undergoing fertility treatments.	4.06

**Table 3.** Sonographer experience survey at the completion of the study, filled per patient (5=completely agree, 4=agree, 3=neutral, 2=disagree, and 1=completely disagree).

Question	Average score
The patient felt physically comfortable while using the home ultrasound device.	3.74
The patient did not experience any discomfort or pain beyond what is typical for a similar vaginal examination conducted in the clinic.	4.09
The instructions provided to the patient during the study were clear enough to perform a successful scan.	3.65
I find it convenient to guide scans remotely with the assistance of a clinician, and the patient cooperated as required during the study.	4.17
I believe the information obtained from the self-scans is of high quality and is sufficient for the necessary treatment and for clinical decisions.	3.78
I would recommend the use of the home device to women undergoing fertility treatments.	4.18

The mean total duration (including preparation time) of the first FC scan was 16.1 (SD 8.2) minutes, and 12.3 (SD 6.03) minutes for the last pretrigger FC scan (*P*=.07).

## Discussion

### Summary

Assisted reproduction imposes a significant burden of repeated vaginal sonographies, resulting in patient stress and substantial loss of time. In this study, we compared the follicular and endometrial measurements obtained from sonographies performed by patients at home with a small portable ultrasound device (FC) with those obtained by standard IC sonographies performed on the same days. The home FC device was found to produce adequate imaging and to be noninferior to the conventional IC measurements in all the clinical parameters significant for assisted reproduction: antral follicle count, follicular measurements of different sizes, endometrial measurements, and correlation with OPU results. Thus, this device may serve as an adequate alternative for IC testing, offering the advantages of home testing and reduced traveling.

### Comparison With Prior Work

The number and size of ovarian follicles and the endometrial thickness as determined by serial sonographies, together with the blood levels of the ovarian steroids and luteinizing hormone, are key parameters for monitoring the female cycle during assisted reproduction treatments [1,2]. The clinical decisions during the cycle, such as initiation of gonadotropin stimulation, the dosage, addition of a gonadotropin-releasing hormone antagonist, and trigger timing, are principally based on the number and size of ovarian follicles [1]. These measurements are performed by serial vaginal sonographies at the fertility clinics or in distal medical sites. Their performance imposes a significant burden of travel time, stress, and cost on both patients and clinics [5]. In clinics with a large patient population, these routine tests, which focus on physiology and not on pathologies, are often performed in a time-restricted manner without full video documentation, precluding the possibility of reviewing the examination.

Previously published studies have aimed to integrate telemedicine into assisted reproduction, advancing its simplicity and accessibility to patients. Gerris et al [4] and Gerris and Fauser [5] have explored the clinical applicability of the use of home ultrasonography by patients or their partners to perform transvaginal sonographies at their convenience in a stepwise manner, thereby significantly alleviating the cost and stress of monitoring. The first study by this group was published in 2009 [9] and demonstrated that patients (n=25) are willing to use home sonography, provided results of the treatment remain unaffected. In 2010, the same group published a proof-of-concept study in a hospital setting, establishing that patients can perform vaginal sonograms themselves (n=20) [8], and in 2014, they published a randomized controlled trial [4] demonstrating that patient-operated home sonography gives similar results (embryological and clinical) and was noninferior to traditional sonographic follow-up. Both patient-reported outcomes and cost were in favor of home sonography (n=121, 59 in the self-operated endovaginal telemonitoring [SOET] arm, and 62 in the non-SOET arm, all expected normal responders) [4]. In 2016, they performed an observational study of intracytoplasmic sperm injection attempts using home sonography that showed just 1 method failure (in 78 patients undergoing 100 consecutive intracytoplasmic sperm injection attempts) [10]. Their main outcome measures were the number of cycles with hospital visits, laboratory and clinical variables (metaphase II oocytes, 2 pronuclear fertilizations, good day-5 blastocysts, pregnancy rate, ongoing pregnancy rate, etc), and transportation avoided, though this study was purely descriptive and did not compare these outcomes to those following IC measurements. Though Gerris et al [4] have studied the use of home ultrasonography, only their randomized controlled trial in 2014 examined the clinical outcomes of home monitoring compared to IC scans. Our study adds to current literature by strengthening their findings and demonstrating the real-life reliability and accuracy of patient self-scans using the smartphone-based FC device, which connects seamlessly with the patient’s personal smartphone, allowing them to perform transvaginal scans of the uterus and ovaries, with remote diagnosis provided by a qualified clinician. Additionally, in our study, correlation coefficients between the FC and IC scans were calculated, providing another measure of accuracy to the home-based monitoring device.

Previous studies have also supported the notion of Gerris et al [4], but with some limitations. For example, a previous prospective study by Resetkova et al [6] evaluated the feasibility of home-based SOET of women undergoing controlled ovarian stimulation with gonadotropins for IVF by comparing it to facility-based testing. This study consisted of 6 subjects, who performed home-based SOET in parallel with standard of care clinic-based ultrasound monitoring of follicle maturation. Participants underwent a 1-hour instructional session involving the use and functionality of the home ultrasound monitoring kit and image acquisition. All participants demonstrated competence in the use of the system before departing the clinic with the kit. Participants performed home-based ultrasounds daily from stimulation day 4 through to the day before oocyte retrieval. A comparative analysis was made at the conclusion of cycle monitoring. They demonstrated that images acquired by home-based SOET correlate well with clinic-based monitoring and that the critical decision to administer a trigger injection compares favorably to clinic-based ultrasound. Objective and subjective measures suggested a high degree of user satisfaction. The strength of our study, compared to theirs, lies in our large sample size, enabling better demonstration of image correlations and examination of patient and physician satisfaction. Another study by Chung et al [7] evaluated the suitability of virtual transvaginal ultrasonography for the evaluation of the ovarian reserve as manifested by the antral follicle count. A crossover trial evaluating ovarian reserve was conducted in 56 women who performed a self-administered virtual transvaginal ultrasonography at home, guided by a remote certified ultrasound technologist, and then underwent an IC transvaginal ultrasonography with another ultrasound technician. The home ultrasound was found to be noninferior to IC transvaginal ultrasonography in producing clinical-quality images and was equivalent for estimating the antral follicle count. However, as opposed to our study, they did not examine home versus IC follicular growth monitoring during an ovarian stimulation cycle for IVF, and thus could not demonstrate the reliability or accuracy of such scans for monitoring these cases.

Another small retrospective study by Dalewyn et al [14] aimed to examine whether there was a correlation in the number of follicles and 2D growth between recordings made using SOET versus measurements performed by a professional sonographer. In this study, 3 different ultrasound moments were recorded and compared in a total of 15 women. At time A, an ultrasound was performed by the patient at home using SOET at the decision time of triggering. At time B, an ultrasound was also recorded by the patient, 24 hours later. At time C, an ultrasound was performed by a physician using a high-end ultrasound device immediately before oocyte retrieval, 12 hours later than time B. The correlation in number and 2D size between the different measurement moments was calculated. They found good correlation in follicle count between time B and C, and that the difference in mean 2D size between different measurement moments was not statistically significant. However, this study, as opposed to ours, did not examine these correlations at the start of ovarian stimulation, thus not examining the true utility (reduced travel time, etc) that home vaginal follicular monitoring may offer to patients undergoing IVF.

These studies all demonstrate that home ultrasound vaginal follicular monitoring is an emerging area of interest, particularly in the context of high ovarian response [15-17], with very few studies (mostly consisting of small sample sizes and partial ovarian stimulation monitoring) actually having examined the real-life reliability and accuracy of patient self-scans using a remote vaginal self-scan device, as compared to standard IC sonographies in patients undergoing an ovarian stimulation cycle for IVF. Therefore, our prospective study adds to clinical knowledge in the field and reinforces the feasibility and accuracy of the use of patient home scans for follicular monitoring.

In our study, we attempted to examine the feasibility of monitoring the ovarian response to gonadotropin stimulation using patient self-scans with a home vaginal device (FC), comparing their results to those obtained by professional sonographers using high-end ultrasound devices (IC) which are considered the gold standard.

The home vaginal device requires patients to have an operational ability to identify and scan their uterus and ovaries with remote guidance. The video clips were then automatically uploaded to the cloud to be read and interpreted by a professional sonographer. Here, we examined the adequacy of the FC clips and measurements in comparison to the IC gold standard in terms of image quality and the correlation of key sonographic parameters in the management of ovarian stimulation. Additionally, we compared the correlation between the follicle numbers obtained by both sonographic modalities (FC and IC) to the actual outcome of the procedure, that is, the numbers of total and mature oocytes picked up.

### Principal Results

The quality score of all images obtained by the FC device was at a minimum suitable for diagnosis, and most of the images obtained had a superior quality score, which was as good as the IC gold standard. The endometrial measurements and the follicular counts and measurements obtained with the FC device were highly correlated with the IC gold standard in all the clinically significant parameters. The antral follicle count, the total number of stimulated follicles, identification when the leading follicle reached 14 mm, and the size of the follicles at the pretriggering visit were all highly correlated between the FC and IC scans. Moreover, we also looked at the relationship between the products of the oocyte aspiration, the total and mature oocyte numbers, and the results of the last FC and IC scans. The ratio between the number of aspirated oocytes per stimulated follicles was highly correlated between the pretriggering FC and IC scans. The same applies to the mature oocyte per follicles >13 mm ratio.

All these findings establish the accuracy of the FC device, its method of operation, and result interpretation as comparable to conventional IC scans and lay the basis for remote home-based monitoring of the follicular development during ovarian stimulation. If such good correlations were obtained during full standard stimulations with multiple follicles for IVF, we believe that similar or even better results could be obtained in monitoring mild stimulations and natural cycles. Thus, similarly, the FC device could be used to reliably monitor the ovarian cycle for the benefit of women who attempt to achieve or avoid a spontaneous conception.

### Limitations and Conclusions

The FC device and this study are not without limitations. Ideally, the FC device should have been evaluated against the IC standard sonography in a prospective randomized controlled trial as 2 totally separate arms in different patients. The current design (FC against IC in the same patients) was chosen due to ethical considerations, taking into account the fact that the FC device has never been evaluated before. Only women with a BMI <40 kg/m^2^ and a normal pelvic anatomy were included. Therefore, our findings may lack generalizability and external validity, particularly for diverse or higher-risk patient populations. For purposes of uniformity, this study was conducted for only 1 stimulation cycle per patient, so no learning or improvement from cycle to cycle was assessed. Nevertheless, the average duration of the last pretrigger FC scans was almost significantly (*P*=.07) shorter than the first scans, even though the ovaries were significantly larger and contained more follicles at the last visit, thus indicating an improvement in patient performance within the same cycle. Additionally, 22% of participants who initially consented to participate in this study found the FC device unsuitable for them during training or this study. This implies that the FC device and operation method do not suit all patients and that proper individual evaluation, selection, and training are required before FC device self-scans can be clinically used. Nevertheless, the burden relief offered by the FC device to patients and clinics is well worth it, even if used only by part of the population. Lastly, the sonographers analyzing the IC scans were not blinded to the source of the data being analyzed, and the results were transferred in real-time directly to the patients’ medical records. On the other hand, the FC scans were analyzed anonymously on a different platform hours after their performance by different sonographers, not aware of each other. Thus, we believe that this separation nullifies any potential interpreter bias. This preliminary study provides valuable insight regarding the potential benefits of the FC self-scans. Despite our prospective study design, future randomized controlled trials comparing different patients from diverse populations performing FC versus IC scans may circumvent some of the limitations mentioned and provide more robust grounds on which to base patient recommendations regarding the use of home vaginal self-scans for ovarian stimulation for IVF, while increasing generalizability.

## Supplementary material

10.2196/72607 Multimedia Appendix 1 Procedure for vaginal self-scans in the clinic by a professional sonographer using the patient interface.

10.2196/72607 Multimedia Appendix 2 Procedure for visualization and scanning of ovaries in the transverse section.

## Abbreviations:

AMB: absolute mean bias
FC: Pulsenmore follicle count home vaginal ultrasound
IC: in-clinic
IVF: in-vitro fertilization
LOA: limits of agreement
OPU: oocyte pick-up
RMSD: root mean square difference
SOET: self-operated endovaginal telemonitoring

## References

[1] Kwan I, Bhattacharya S, Woolner A (2021). Monitoring of stimulated cycles in assisted reproduction (IVF and ICSI). Cochrane Database Syst Rev.

[2] Simeonov M, Sapir O, Lande Y (2020). The entire range of trigger-day endometrial thickness in fresh IVF cycles is independently correlated with live birth rate. Reprod Biomed Online.

[3] Bedient CE, Kaye LA, Raman A, Garner FC, Aguirre M, Shapiro BS (2019). The optimal size of ovarian follicles at oocyte collection. Fertil Steril.

[4] Gerris J, Delvigne A, Dhont N (2014). Self-operated endovaginal telemonitoring versus traditional monitoring of ovarian stimulation in assisted reproduction: an RCT. Hum Reprod.

[5] Gerris JMR, Fauser BCJM (2020). Home monitoring of ovarian stimulation: an important step towards more patient-centred IVF. Reprod Biomed Online.

[6] Resetkova N, Sakkas D, Bayer S, Penzias A, Alper MM (2016). Home-based ultrasound monitoring for in vitro fertilization is a feasible method of in cycle monitoring. Fertil Steril.

[7] Chung EH, Petishnok LC, Conyers JM (2022). Virtual compared with in-clinic transvaginal ultrasonography for ovarian reserve assessment. Obstet Gynecol.

[8] Gerris J, De Sutter P (2010). Self-operated endovaginal telemonitoring (SOET): a step towards more patient-centred ART?. Hum Reprod.

[9] Gerris J, Geril A, De Sutter P (2009). Patient acceptance of self-operated endovaginal telemonitoring (SOET): proof of concept. Facts Views Vis Obgyn.

[10] Gerris J, Vandekerckhove F, De Sutter P (2016). Outcome of one hundred consecutive ICSI attempts using patient operated home sonography for monitoring follicular growth. Facts Views Vis Obgyn.

[11] Alviggi C, Andersen CY, Poseidon Group (Patient-Oriented Strategies Encompassing Individualized Oocyte Number) (2016). A new more detailed stratification of low responders to ovarian stimulation: from a poor ovarian response to a low prognosis concept. Fertil Steril.

[12] (2018). Emergency ultrasound standard reporting guidelines. https://www.acep.org/siteassets/uploads/uploaded-files/acep/clinical-and-practice-management/policy-statements/information-papers/emergency-ultrasound-standard-reporting-guidelines---2018.pdf.

[13] Mahajan N, Sharma S (2016). The endometrium in assisted reproductive technology: how thin is thin?. J Hum Reprod Sci.

[14] Dalewyn L, Deschepper E, Gerris J (2017). Correlation between follicle dimensions recorded by patients at home (SOET) versus ultrasound performed by professional care providers. Facts Views Vis Obgyn.

[15] Drakopoulos P, Khalaf Y, Esteves SC (2023). Treatment algorithms for high responders: what we can learn from randomized controlled trials, real-world data and models. Best Pract Res Clin Obstet Gynaecol.

[16] Robertson I, Chmiel FP, Cheong Y (2021). Streamlining follicular monitoring during controlled ovarian stimulation: a data-driven approach to efficient IVF care in the new era of social distancing. Hum Reprod.

[17] Liang X, Liang J, Zeng F (2022). Evaluation of oocyte maturity using artificial intelligence quantification of follicle volume biomarker by three-dimensional ultrasound. Reprod Biomed Online.

